# Development and Validation of a Deep Learning–Based Automated Detection Algorithm for Major Thoracic Diseases on Chest Radiographs

**DOI:** 10.1001/jamanetworkopen.2019.1095

**Published:** 2019-03-22

**Authors:** Eui Jin Hwang, Sunggyun Park, Kwang-Nam Jin, Jung Im Kim, So Young Choi, Jong Hyuk Lee, Jin Mo Goo, Jaehong Aum, Jae-Joon Yim, Julien G. Cohen, Gilbert R. Ferretti, Chang Min Park

**Affiliations:** 1 Department of Radiology, Seoul National University College of Medicine, Seoul, South Korea; 2 Lunit Inc, Seoul, South Korea; 3 Department of Radiology, Seoul National University Boramae Medical Center, Seoul, South Korea; 4 Department of Radiology, Kyung Hee University Hospital at Gangdong, Kyung Hee University College of Medicine, Seoul, South Korea; 5 Department of Radiology, Eulji University Medical Center, College of Medicine, Seoul, South Korea; 6 Division of Pulmonary and Critical Care Medicine, Department of Internal Medicine, Seoul National University College of Medicine, Seoul, South Korea; 7 Pôle Imagerie, Centre Hospitalier Universitaire de Grenoble, La Tronche, France

## Abstract

**Importance:**

Interpretation of chest radiographs is a challenging task prone to errors, requiring expert readers. An automated system that can accurately classify chest radiographs may help streamline the clinical workflow.

**Objectives:**

To develop a deep learning–based algorithm that can classify normal and abnormal results from chest radiographs with major thoracic diseases including pulmonary malignant neoplasm, active tuberculosis, pneumonia, and pneumothorax and to validate the algorithm’s performance using independent data sets.

**Design, Setting, and Participants:**

This diagnostic study developed a deep learning–based algorithm using single-center data collected between November 1, 2016, and January 31, 2017. The algorithm was externally validated with multicenter data collected between May 1 and July 31, 2018. A total of 54 221 chest radiographs with normal findings from 47 917 individuals (21 556 men and 26 361 women; mean [SD] age, 51 [16] years) and 35 613 chest radiographs with abnormal findings from 14 102 individuals (8373 men and 5729 women; mean [SD] age, 62 [15] years) were used to develop the algorithm. A total of 486 chest radiographs with normal results and 529 with abnormal results (1 from each participant; 628 men and 387 women; mean [SD] age, 53 [18] years) from 5 institutions were used for external validation. Fifteen physicians, including nonradiology physicians, board-certified radiologists, and thoracic radiologists, participated in observer performance testing. Data were analyzed in August 2018.

**Exposures:**

Deep learning–based algorithm.

**Main Outcomes and Measures:**

Image-wise classification performances measured by area under the receiver operating characteristic curve; lesion-wise localization performances measured by area under the alternative free-response receiver operating characteristic curve.

**Results:**

The algorithm demonstrated a median (range) area under the curve of 0.979 (0.973-1.000) for image-wise classification and 0.972 (0.923-0.985) for lesion-wise localization; the algorithm demonstrated significantly higher performance than all 3 physician groups in both image-wise classification (0.983 vs 0.814-0.932; all *P* < .005) and lesion-wise localization (0.985 vs 0.781-0.907; all *P* < .001). Significant improvements in both image-wise classification (0.814-0.932 to 0.904-0.958; all *P* < .005) and lesion-wise localization (0.781-0.907 to 0.873-0.938; all *P* < .001) were observed in all 3 physician groups with assistance of the algorithm.

**Conclusions and Relevance:**

The algorithm consistently outperformed physicians, including thoracic radiologists, in the discrimination of chest radiographs with major thoracic diseases, demonstrating its potential to improve the quality and efficiency of clinical practice.

## Key Points

**Question:**

Can a deep learning–based algorithm accurately discriminate abnormal chest radiograph results showing major thoracic diseases from normal chest radiograph results?

**Findings:**

In this diagnostic study of 54 221 chest radiographs with normal findings and 35 613 with abnormal findings, the deep learning–based algorithm for discrimination of chest radiographs with pulmonary malignant neoplasms, active tuberculosis, pneumonia, or pneumothorax demonstrated excellent and consistent performance throughout 5 independent data sets. The algorithm outperformed physicians, including radiologists, and enhanced physician performance when used as a second reader.

**Meaning:**

A deep learning–based algorithm may help improve diagnostic accuracy in reading chest radiographs and assist in prioritizing chest radiographs, thereby increasing workflow efficacy.

## Teaser

This diagnostic study measures the ability of a deep learning–based algorithm to discriminate between chest radiographs with normal results and chest radiographs with abnormal results indicating major thoracic disease.

## Introduction

Chest radiographs (CRs) have been used as a first-line examination for the evaluation of various thoracic diseases worldwide.^1,2^ In fact, CR is the most commonly performed radiologic examination today, accounting for up to 26% of all diagnostic radiologic examinations.^3^ Interpretation of CR, however, remains a challenging task requiring both experience and expertise, as various anatomic structures can overlap in a single 2-dimensional image, and various physiological and pathological changes may appear similar or a single pathology may exhibit various features.^4^ Thus, interpretation is prone to errors, with a previous study^5^ reporting that 22% of all errors in diagnostic radiology were made in CRs. Compounding this difficulty is an increase in the number of examinations at a rate much faster than the increase in the number of qualified radiologists, which has led to an increased workload for radiologists.^6^

Thus, it is not surprising that computer-aided diagnosis (CAD) for CRs has remained an attractive topic for researchers.^7^ Indeed, there have been several CAD systems that have achieved successful results for various thoracic diseases, including pulmonary nodules,^8,9^ pulmonary tuberculosis,^10,11^ and pneumothorax.^12^ However, few CAD systems are presently used in clinical practice owing to their suboptimal performance (sensitivity, 47%-76% with 1.7-3.3 false-positives per image for lung nodules; area under the receiver operating characteristic curve [AUROC], 0.71-0.84 for pulmonary tuberculosis),^10,13^ and specific target diseases limit their utility in general practice.

Recently, the deep learning technique demonstrated promising results in medical image analyses, including detecting diabetic retinopathy in fundus photographs,^14^ classifying skin cancer from skin photographs,^15^ and detecting metastasis on pathologic images.^16^ As for CRs, several studies have reported notable performance of the deep learning algorithm for image classification.^17,18,19^ However, algorithms in those studies have not yet been fully validated in unseen data sets,^17,18,19^ limiting the generalizability of results.

Previously, we investigated deep learning–based automatic detection algorithms (DLADs) for classification of CRs with malignant nodules^20^ and active pulmonary tuberculosis.^21^ However, those algorithms had limited clinical utility, as there are various pathologies and abnormalities other than malignant neoplasms and pulmonary tuberculosis in real-world clinical practice. For a CAD system to have practical value in this setting, it should work on CRs with various abnormalities, particularly major thoracic diseases that account for most thoracic abnormalities observed on CRs. Therefore, the purpose of our study was to develop a DLAD for major thoracic diseases on CRs and to validate its performance using independent data sets in comparison with physicians.

## Methods

This study was approved by the institutional review boards of all participating institutions, which waived the requirement for patient consent. This report followed the Standards for Reporting of Diagnostic Accuracy (STARD) 2015 reporting guideline.

Among the development data set, CRs with normal findings (n = 54 221) and some CRs with abnormal findings of pulmonary malignant neoplasms (n = 13 926) and active pulmonary tuberculosis (n = 6768) were used in our previous studies.^20,21^ In those studies, however, tasks of the developed algorithms were classification of CRs with pulmonary malignant neoplasms^20^ and active pulmonary tuberculosis from normal CRs, which is different from that of our current study.^21^

### Definition of Target Diseases and the Primary Task of the DLAD

We defined the target diseases of our DLAD as major thoracic diseases that are common, clinically important, and detectable on CRs. Specifically, we included pulmonary malignant neoplasms (including primary lung cancers and metastasis), active pulmonary tuberculosis, and pneumonia, which are among the top 5 respiratory diseases in terms of global burden.^22,23^ We added pneumothorax as a target disease, as it is relatively common, yet can cause mortality without prompt and accurate detection through CRs.^24,25^

The primary goal of our DLAD was binary classification of CRs: CRs with abnormal findings including any of the target diseases vs normal CRs. The subsidiary goal of our DLAD was to differentiate CRs with abnormal results into 1 of 4 major thoracic diseases.

### Development of the DLAD

#### Data Collection and Curation

For the development of the DLAD, a total of 57 481 CRs with normal results and 41 140 CRs with abnormal results were retrospectively collected between November 1, 2016, and January 31, 2017, from a single institution (institution A). The CRs with normal findings were collected based on their radiology reports and were double-checked by board-certified radiologists. The CRs with abnormal findings were obtained from patients with pathologically proven or clinically and/or radiologically confirmed diseases, of which the detailed inclusion criteria are summarized in eTable 1 in the Supplement. Abnormal findings of CRs included the following 4 disease categories: pulmonary malignant neoplasms, active pulmonary tuberculosis, pneumonia, and pneumothorax.

For data curation, all CRs were reviewed by at least 1 of 15 board-certified radiologists (7-14 years of experience in reading CRs). The data curation process comprised 2 steps. The first step, image labeling, was performed to confirm whether each CR was categorized correctly and whether abnormalities of CRs with abnormal results were visible on the CRs. The second step, image annotation, marked the exact location of each abnormal finding on the CR. During data curation, CRs originally designated as normal but showing significant abnormality (3260 CRs) and CRs read as abnormal but without detectable abnormal finding (5527 CRs) were excluded from the data set by reviewing radiologists. Finally, 54 221 CRs with normal results from 47 917 individuals (21 556 men and 26 361 women; mean [SD] age, 51 [16] years) and 35 613 CRs with abnormal results from 14 102 individuals (8373 men and 5729 women; mean [SD] age, 62 [15] years) were used for the development of the DLAD. Annotations were performed in 35.6% of CRs with abnormal results (12 696 of 35 613).

All CRs were randomly assigned into 1 of the 3 following data sets: (1) training data set comprising 53 621 CRs with normal findings and 34 074 CRs with abnormal findings to optimize network weights; (2) tuning data set comprising 300 CRs with normal findings and 750 CRs with abnormal findings to optimize hyperparameters; and (3) in-house validation data set comprising 300 CRs with normal findings and 789 CRs with abnormal findings to evaluate the detection performance of the trained DLAD (eFigure 1 in the Supplement). To prevent CRs of a single individual from being assigned to different data sets, patient-based assignment was performed, and the patients of the 3 data sets were excluded from the other data sets.

#### Development of the DLAD Algorithm

Detailed description of the network architecture and the training of the DLAD is provided in the eAppendix and eFigure 2 in the Supplement. In brief, we adopted a deep convolutional neural network with dense blocks^26^ comprising 5 parallel classifiers. Four classifiers were designed for each disease, and the final classifier was designed for classification of CRs with abnormal results reflecting any of the target diseases. To train the algorithm to classify abnormal CRs with major thoracic diseases as well as to localize abnormalities, 2 types of losses were used to train the algorithm: classification loss and localization loss. Both CRs with and without annotations were used in training, while localization losses were calculated only from the CRs with annotations.

Finally, for each input CR, the DLAD provided continuous value between 0 and 1 as the image-level probability of abnormal CR. Per-pixel localization probability maps for each target disease and the entire target disease, overlaid on the input CR, were also provided.

#### Evaluation of DLAD Performance

First, the performance of the DLAD was evaluated using an in-house validation data set, part of the development data set not used for training. Thereafter, external validation tests were performed using 5 independent data sets separately collected and curated between May 1 and July 31, 2018 at different institutions (4 hospitals in Korea [institutions A-D] and 4 hospital in France [institution E]) to validate the consistency of the DLAD’s performance. The inclusion criteria for the external validation data sets are summarized in eTable 1 in the Supplement. Each CR with abnormal results contained only 1 disease finding of the 4 target diseases. All CRs with normal and abnormal findings, except CRs with pneumothorax, had corresponding chest computed tomographic images to define firm reference standards. Five board-certified radiologists (7-14 years of experience) in each of the participating institutions selected and labeled the CRs and annotated the exact locations of the abnormalities on each CR with abnormal results. The external validation data set from institution A was temporally different from the development data set. Demographic information regarding the external validation data sets appears in eTable 2 in the Supplement. Overall, a total of 486 CRs with normal results and 529 with abnormal results (1 from each participant; 628 men and 387 women; mean [SD] age, 53 [18] years) were used for external validation.

### Observer Performance Test

To compare the performances between the DLAD and physicians and to evaluate whether a DLAD can improve physicians’ diagnostic performance, an observer performance test was conducted. An observer panel of 15 physicians with varying experience (5 thoracic radiologists [9-14 years of experience]; 5 board-certified radiologists [5-7 years of experience]; and 5 nonradiology physicians) was constructed. For this test, the external validation data set from institution A was used. The radiologist who defined the reference standard for the data set did not participate in the test. The test included 2 sessions. In session 1, observers independently assessed every CR, without assistance of the DLAD, to classify CRs into those with significant abnormal findings requiring treatment or further evaluation and those without. Observers were also asked to localize the abnormal finding via free-hand annotation, along with a confidence score of a continuous value between 0 and 1 for each annotation. In session 2, observers reevaluated every CR with the assistance of the DLAD and were asked to modify their original decision if necessary (eFigure 3 in the Supplement).

### Statistical Analysis

All statistical analyses were performed in August 2018 using R statistical software version 3.5.1 (R Project for Statistical Computing)^27^ with package RJafroc.^28^ Receiver operating characteristic (ROC) analyses and jackknife alternative free-response ROC (JAFROC) analyses were performed to evaluate image-wise classification and lesion-wise localization performances, respectively. For the DLAD, the image-wise probability value of each CR and the maximum pixel-wise probability value in the predefined reference standard annotation were considered to be confidence scores for ROC and JAFROC analyses, respectively. For physicians, the highest confidence score among annotated lesions in each image was used as their confidence score for image-wise classification.^29^ The AUROCs and area under the alternative free-response ROC curve (AUAFROCs) were used as performance measures of ROC and JAFROC analyses, respectively. Statistical significances were evaluated using a method suggested by Dorfman et al.^30^ Both the observers and cases were treated as random effects for analyses in the physician groups, while only cases were treated as random effects for analyses in individual physicians.^31^

In addition, sensitivities, specificities, precision, and F1 scores for image-wise classification were evaluated. For the DLAD, 2 different probability values were selected as classification thresholds based on the results of in-house validation: a high sensitivity threshold (95% sensitivity on in-house validation) and a high specificity threshold (95% specificity on in-house validation). For physicians, any detected lesion was regarded to be positive. Comparison of sensitivities and specificities were performed using McNemar tests.

For performance evaluation in differentiating CRs with abnormal findings, the disease with the highest probability value among the DLAD’s output was regarded as the differential diagnosis of the DLAD. Thereafter, confusion matrices were drawn and overall accuracies and producer’s accuracies of each target disease were calculated.^32^

All results with 2-sided *P* values less than .05 were considered to indicate a statistically significant difference, and the Holm-Bonferroni method was used to correct for multiple comparisons.^33^

## Results

### Image-Wise Classification Performance of the DLAD

The performance of the DLAD in our in-house validation data set revealed an AUROC of 0.965 (95% CI, 0.955-0.975) for image-wise classification. From this result, operating thresholds were defined as a probability of 0.16 (high sensitivity threshold; sensitivity, 0.951; specificity, 0.750) and 0.46 (high specificity threshold; sensitivity, 0.920; specificity, 0.950).

**Figure 1.**
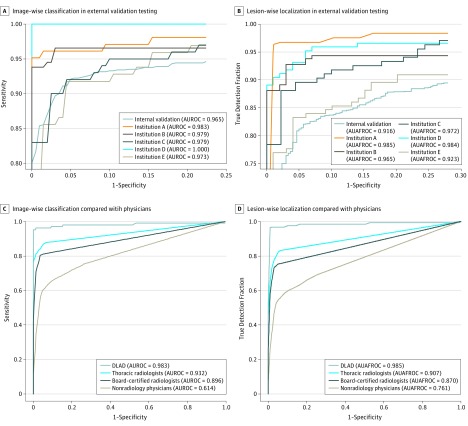
Results of External Validation Tests and Observer Performance Tests The deep learning–based automatic detection algorithm (DLAD) showed consistently high image-wise classification (area under the receiver operating characteristic curve [AUROC], 0.973-1.000) (A) and lesion-wise localization (area under the alternative free-response receiver operating characteristic curve [AUAFROC], 0.923-0.985) (B) performances in external validation tests. In comparison of performance with physicians, the DLAD showed significantly high classification (AUROC, 0.983 vs 0.814-0.932) (C) and localization (AUAFROC, 0.985 vs 0.781-0.907) (D) performances than all observer groups.

For external validation, the DLAD showed a median (range) AUROC of 0.979 (0.973-1.000), which was greater than the results of in-house validation. Median (range) sensitivity and specificity were 0.979 (0.913-1.000) and 0.880 (0.566-1.000), respectively, using the high sensitivity threshold; median (range) sensitivity and specificity were 0.945 (0.845-1.000) and 0.980 (0.848-1.000), respectively, using the high specificity threshold. For individual diseases, sensitivities of the DLAD were between 0.833 and 1.000 using the high sensitivity threshold, and between 0.808 and 1.000 using the high specificity threshold (eTable 3 in the Supplement). Detailed performances are described in Table 1 and Figure 1.

**Table 1.** Performance of the Deep Learning–Based Automatic Detection Algorithm in the 5 External Validation Tests

Measure	Performance (95% CI)				
	Institution				
	A	B	C	D	E
AUROC	0.983 (0.961-1.004)	0.979 (0.960-0.998)	0.979 (0.962-0.996)	1.000 (1.000-1.000)	0.973 (0.949-0.996)
AUAFROC	0.985 (0.967-1.004)	0.965 (0.941-0.989)	0.972 (0.953-0.990)	0.984 (0.971-0.997)	0.923 (0.879-0.967)
High sensitivity threshold					
Sensitivity	0.913 (0.841-0.959)	0.973 (0.931-0.992)	1.000 (0.964-1.000)	1.000 (0.957-1.000)	0.979 (0.927-0.997)
Specificity	1.000 (0.963-1.000)	0.880 (0.800-0.936)	0.633 (0.525-0.732)	0.940 (0.874-0.978)	0.566 (0.462-0.665)
Precision	1.000 (0.962-1.000)	0.922 (0.868-0.959)	0.752 (0.670-0.823)	0.933 (0.825-0.948)	0.688 (0.604-0.764)
F1 score	0.955 (0.897-0.979)	0.947 (0.898-0.975)	0.858 (0.791-0.903)	0.965 (0.886-0.973)	0.808 (0.731-0.865)
High specificity threshold					
Sensitivity	0.845 (0.760-0.909)	0.945 (0.895-0.976)	0.970 (0.915-0.994)	1.000 (0.957-1.000)	0.918 (0.844-0.964)
Specificity	1.000 (0.963-1.000)	0.980 (0.930-0.998)	0.878 (0.792-0.937)	1.000 (0.964-1.000)	0.848 (0.762-0.913)
Precision	1.000 (0.959-1.000)	0.986 (0.949-0.998)	0.898 (0.825-0.948)	1.000 (0.957-1.000)	0.856 (0.773-0.917)
F1 score	0.916 (0.848-0.952)	0.965 (0.921-0.987)	0.933 (0.868-0.970)	1.000 (0.957-1.000)	0.886 (0.807-0.940)

Abbreviations: AUAFROC, area under the alternative free-response receiver operating characteristic curve; AUROC, area under the receiver operating characteristic curve.

### Lesion-Wise Localization Performance of the DLAD

In-house performance of the DLAD in lesion-wise localization was an AUAFROC of 0.916 (95% CI, 0.900-0.932). In the external validation data sets, the DLAD showed a median (range) AUAFROC of 0.972 (0.923-0.985) (Table 1 and Figure 1).

### Comparison Between the DLAD and Physicians

In session 1 of the observer performance test, pooled AUROCs for nonradiology physicians, board-certified radiologists, and thoracic radiologists were 0.814, 0.896, and 0.932, respectively. The performance of the DLAD (AUROC 0.983) was significantly higher than all 3 observer groups (All *P* < .005) (Table 2 and Figure 1).

**Table 2.** Performance of Physicians in the Observer Performance Test

Observer Group	AUROC (95% CI)	*P* Value	AUAFROC (95% CI)	P Value	Sensitivity (95% CI)	*P* Value	Specificity (95% CI)	*P* Value
**Session 1 (Physician Without DLAD Assistance)**								
Nonradiology physicians	0.814 (0.764-0.864)	<.001^a^	0.781 (0.731-0.832)	<.001^a^	0.699 (0.657-0.738)	NA	0.901 (0.871-0.926)	NA
Board-certified radiologists	0.896 (0.856-0.937)	<.001^a^	0.870 (0.830-0.910)	<.001^a^	0.812 (0.775-0.845)	NA	0.948 (0.925-0.966)	NA
Thoracic radiologists	0.932 (0.901-0.963)	.002^a^	0.907 (0.874-0.940)	<.001^a^	0.876 (0.844-0.903)	NA	0.946 (0.922-0.965)	NA
**Session 2 (Physician With DLAD Assistance)**								
Nonradiology physicians	0.904 (0.852-0.957)	<.001^b^	0.873 (0.815-0.931)	<.001^b^	0.835 (0.800-0.866)	<.001^b^	0.924 (0.896-0.946)	.006^b^
Board-certified radiologists	0.939 (0.911-0.968)	<.001^b^	0.919 (0.886-0.951)	<.001^b^	0.893 (0.863-0.919)	<.001^b^	0.948 (0.925-0.966)	.62^b^
Thoracic radiologists	0.958 (0.935-0.982)	.002^b^	0.938 (0.914-0.961)	<.001^b^	0.924 (0.898-0.946)	<.001^b^	0.948 (0.925-0.966)	>.99^b^

Abbreviations: AUAFROC, area under the alternative free-response receiver operating characteristic curve; AUROC, area under the receiver operating characteristic curve; DLAD, deep learning–based automatic detection algorithm; NA, not applicable.

^a^ Comparison of performance with DLAD.

^b^ Comparison of performance with session 1.

For the lesion-wise localization, pooled AUAFROCs for nonradiology physicians, board-certified radiologists, and thoracic radiologists were 0.781, 0.870, and 0.907, respectively. The performance of the DLAD (AUAFROC 0.985) was significantly higher than all observer groups (All *P* < .001).

Regarding the performances of individual observers, the DLAD showed significantly better image-wise classification performance than 14 of 15 observers (median [range] AUROC, 0.906 [0.779-0.959]), and significantly better lesion-wise localization performance than all observers (median [range] AUAFROC, 0.877 [0.742-0.938]) (eTables 4-6 in the Supplement).

### Comparison Between Physician-Only Reading and Physician Assisted by the DLAD

In session 2 of the observer performance test, AUROCs of nonradiology physicians, board-certified radiologists, and thoracic radiologists were 0.904, 0.939, and 0.958, respectively. Increments of AUROCs were 0.090, 0.043, and 0.026, respectively, all of which were statistically significant (all *P* < .005) (Table 2; eFigure 4 in the Supplement).

For lesion-wise localization, AUAFROCs of nonradiology physicians, board-certified radiologists, and thoracic radiologists were 0.873, 0.919, and 0.938, respectively. Increments of AUAFROCs were 0.092, 0.049, and 0.031, respectively, all of which were statistically significant (all *P* < .001).

In terms of sensitivities and specificities, significant improvement in sensitivities (0.699-0.876 in session 1; 0.835-0.924 in session 2; all *P* < .001) were observed in all 3 physician groups, while specificities (0.901-0.946 in session 1; 0.924-0.948 in session 2) were significantly improved only in nonradiology physicians.

In terms of individual observers, significant improvements in AUROCs (median [range] increment, 0.040 [0.007-0.111]) and AUAFROCs (median [range] increment, 0.051 [0.015-0.108]) were observed in 14 of 15 physicians (eTables 4-6 in the Supplement).

**Figure 2.**
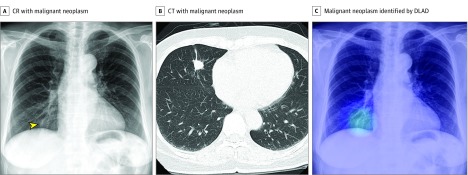
Representative Case From the Observer Performance Test (Malignant Neoplasm) A, The chest radiograph (CR) shows nodular opacity at the right lower lung field (arrowhead), which was initially detected by 2 of 15 observers. B, The corresponding computed tomographic (CT) image reveals a nodule at the right middle lobe. C, The deep learning–based automatic detection algorithm (DLAD) correctly localized the lesion (probability score, 0.291). Four observers additionally detected the lesion after checking the output.

**Figure 3.**
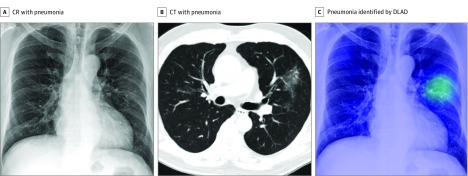
Representative Case From the Observer Performance Test (Pneumonia) A, The chest radiograph (CR) shows subtle patchy increased opacity at the left middle lung field, which was initially missed by all 15 observers. B, The corresponding computed tomographic (CT) image shows patchy ground glass opacity at the left upper lobe. C, The deep learning–based automatic detection algorithm (DLAD) correctly localized the lesion (probability score, 0.371). Seven observers correctly detected the lesion after checking the result.

Figure 2, Figure 3, and eFigure 5 and eFigure 6 in the Supplement show representative images from the observer performance test.

### Differentiation of CRs With Abnormal Findings

In the evaluation of the DLAD’s performance in differentiating CRs with abnormal findings, the pooled overall accuracy was 0.686 (95% CI, 0.645-0.726), while the producer’s accuracies for pulmonary malignant neoplasm, active pulmonary tuberculosis, pneumonia, and pneumothorax were 0.840 (95% CI, 0.774-0.892), 0.209 (95% CI, 0.143-0.290), 0.731 (95% CI, 0.642-0.808), and 0.950 (95% CI, 0.893-0.981), respectively. Detailed results are shown in eTable 7, eTable 8, and eFigures 7 to 9 in the Supplement.

## Discussion

We developed a DLAD algorithm that is able to classify CRs with normal vs abnormal findings indicative of major thoracic diseases including pulmonary malignant neoplasms, active pulmonary tuberculosis, pneumonia, and pneumothorax. Our algorithm consistently demonstrated high performance across independent data sets, even outperforming physicians, including thoracic radiologists. Furthermore, we demonstrated improved physician performance with the assistance of the DLAD.

The strengths of our study can be summarized as follows. First, the development data set underwent extensive data curation by radiologists. It has been shown that the performance of deep learning–based algorithms depends not only on the quantity of the training data set, but also on the quality of the data labels.^34^ As for CRs, several open-source data sets are currently available; however, those data sets remain suboptimal for the development of deep learning–based algorithms because they are weakly labeled by radiologic reports^35^ or lack localization information.^36^ In contrast, in the present study, we initially collected data from the radiology reports and clinical diagnosis; then experienced board-certified radiologists meticulously reviewed all of the collected CRs. Furthermore, annotation of the exact location of each abnormal finding was done in 35.6% of CRs with abnormal results, which we believe led to the excellent performance of our DLAD.^37^

Second, our DLAD showed consistently excellent performance in the 5 external validation data sets, each of which was independently collected from different institutions across different continents (AUROC, 0.973-1.000; AUAFROC, 0.923-0.985). This consistent performance of the DLAD across the external validation data sets acquired from different populations suggests that our DLAD’s performance may be generalized to various populations.

Third, we compared the performance of our DLAD with the performance of physicians with various levels of experience. The stand-alone performance of a CAD system can be influenced by the difficulty of the test data sets and can be exaggerated in easy test data sets. However, observer performance tests may provide a more objective measure of performance by comparing the performance between the CAD system and physicians. Impressively, the DLAD demonstrated significantly higher performance both in image-wise classification and lesion-wise localization than all physician groups, even the thoracic radiologist group.

Fourth, our DLAD provided localization information as well as image-wise classification capabilities. Although the exact localization of abnormalities on CR may not be a clinically relevant task, it may be an important consideration in the reliability of an algorithm. The explainable output of deep learning algorithms can be critical for the reliability of the algorithms, particularly in the medical field.^38^ Localization information provided by the DLAD can help visualize the logical background of the classification output, which is the ultimate goal of our DLAD. Indeed, the improvement of physicians’ performances with the assistance of the DLAD suggests that it provides a reliable explanation.

The primary goal of our DLAD was to classify CRs with normal vs abnormal results indicating any of the major thoracic diseases. In most clinical situations, CRs serve as the initial diagnostic examination for various thoracic diseases. Detection of such abnormalities would lead to further diagnostic workups with other radiologic or laboratory examinations to make a specific diagnosis. Therefore, the initial detection of such clinically relevant abnormalities is of paramount importance in the interpretation of CRs in real clinical practice. The target diseases of our DLAD, although they did not cover all of the thoracic diseases, were the most common, clinically relevant diseases. Pulmonary malignant neoplasm, tuberculosis, and pneumonia, which are responsible for 1.6, 1.4, and 4 million global deaths per year, respectively, are 3 of the most important diseases among all thoracic diseases that can be detected on CRs.^23^ While it causes less mortality than the 3 other target diseases, pneumothorax is still an important global health burden, with an annual incidence of 18 to 28 and 1.2 to 6 cases per 100 000 males and females, respectively.^39^ Furthermore, it is critical to detect pneumothorax on CRs, as CRs are typically the final diagnostic examination to confirm the diagnosis.

The high performance of the DLAD in classification of CRs with normal and abnormal findings indicative of major thoracic diseases, outperforming even thoracic radiologists, suggests its potential for stand-alone use in select clinical situations. It may also help improve the clinical workflow by prioritizing CRs with suspicious abnormal findings requiring prompt diagnosis and management. It can also improve radiologists’ work efficiency, which would partially alleviate the heavy workload burden that radiologists face today and improve patients’ turnaround time. Furthermore, the improved performance of physicians with the assistance of the DLAD indicates the potential of our DLAD as a second reader. The DLAD can contribute to reducing perceptual error of interpreting physicians by alerting them to the possibility of major thoracic diseases and visualizing the location of the abnormality. In particular, the more obvious increment of performance in less-experienced physicians suggests that our DLAD can help improve the quality of CR interpretations in situations in which expert thoracic radiologists may not be available.

Providing a differential diagnosis among the CRs with abnormal results was a subsidiary task of our DLAD. According to our study results, the DLAD showed promising but suboptimal performance in this task (pooled overall accuracy of 0.686). Actually, one of the most important challenges in the interpretation of CRs is that there are substantial overlaps between the radiological findings of various diseases. Therefore, it is often impossible to provide a specific differential diagnosis using only CRs. As expected, our DLAD showed substantial misclassification among pulmonary malignant neoplasms, tuberculosis, and pneumonia owing to these overlaps in radiologic findings. For pneumothorax, on the other hand, which is one of the few examples in which a specific diagnosis can be made with CRs alone because of the condition’s clearly different findings from other diseases, the DLAD showed excellent differentiating performance (pooled producer’s accuracy of 0.950).

### Limitations

There are several limitations in the present study. First, validation was performed using experimentally designed data sets; however, real-world situations may be substantially different from these data sets, particularly regarding disease prevalence and diversity of abnormalities on CRs. In this regard, further validation and clinical utility tests in various clinical settings are warranted. Second, our DLAD covers only 4 major thoracic disease categories. However, we believe our DLAD algorithm can detect a substantial proportion of clinically relevant diseases in actual practice. At present, training our DLAD to detect all kinds of thoracic diseases, including rare or clinically irrelevant abnormalities, may not be practical because it can cause many false-positive classifications, hampering its clinical utility. Third, each abnormal CR in the external validation data sets represented only 1 target disease, as we attempted to set a strict reference standard for CRs with abnormal findings. However, CRs with multiple target diseases are not uncommon in real-world situations and, thus, warrant future investigations.

## Conclusions

We developed a DLAD algorithm that can classify CRs with normal and abnormal findings indicating major thoracic diseases with consistently high performance, outperforming even radiologists, which may improve the quality and efficiency of the current clinical workflow.

## Notes

Supplement. **eAppendix.** Supplemental Material **eReferences** **eFigure 1.** Flow Diagram of Data Inclusion, Curation, and Allocation **eFigure 2.** Architecture of the DLAD Algorithm **eFigure 3.** User Interface of the Observer Performance Test **eFigure 4.** Comparison of Performances Between Physician Only Reading and Physician Assisted by DLAD **eFigure 5.** Representative Case From the Observer Performance Test (Active Pulmonary Tuberculosis) **eFigure 6.** Representative Case From the Observer Performance Test (Pneumothorax) **eFigure 7.** Confusion Matrices for Differentiation of Abnormal CRs **eFigure 8.** Examples of the Differentiation of Abnormal CRs by DLAD **eFigure 9.** Examples of Differentiation of False-Positively Classified Normal CRs by DLAD **eTable 1.** Inclusion and Exclusion Criteria for the Development and External Validation Datasets **eTable 2.** Demographic Description of the Five External Validation Datasets **eTable 3.** Sensitivities of DLAD for Individual Diseases in the 5 External Validation Datasets **eTable 4.** Performance of Individual Non-Radiology Physician Readers **eTable 5.** Performance of Individual Board-Certified Radiologist Readers **eTable 6.** Performance of Individual Thoracic Radiologist Readers **eTable 7.** Performance of DLAD in the Differentiation of Abnormal CRs **eTable 8.** Differentiation of False-Positively Classified Normal CRs by DLAD
