# Error in Table, Methods Section, and Supplement

**DOI:** 10.1001/jamanetworkopen.2019.3260

**Published:** 2019-04-12

In the Original Investigation titled “Development and Validation of a Deep Learning–Based Automated Detection Algorithm for Major Thoracic Diseases on Chest Radiographs,”^1^ published March 22, 2019, Table 1 and the Methods section referred to the “recall” of the model but should have referred to the “precision” of the model. In addition, eFigure 1 in the Supplement listed incorrect numbers of CRs and patients in the “Inappropriate label” box for total abnormal as well as in the “Training dataset” box for malignancy. This article has been corrected.^1^
