# Supplementary material for: Development and Validation of a Deep Learning–Based Automated Detection Algorithm for Major Thoracic Diseases on Chest Radiographs
Source: JAMA Netw Open. 2019 Mar 22;2(3):e191095. doi: 10.1001/jamanetworkopen.2019.1095 (PMC6583308; doi:10.1001/jamanetworkopen.2019.1095)
Supplement: Supplement. — eAppendix. Supplemental Material eReferences eFigure 1. Flow Diagram of Data Inclusion, Curation, and Allocation eFigure 2. Architecture of the DLAD Algorithm eFigure 3. User Interface of the Observer Performance Test eFigure 4. Comparison of Performances Between Physician Only Reading and Physician Assisted by DLAD eFigure 5. Representative Case From the Observer Performance Test (Active Pulmonary Tuberculosis) eFigure 6. Representative Case From the Observer Performance Test (Pneumothorax) eFigure 7. Confusion Matrices for Differentiation of Abnormal CRs eFigure 8. Examples of the Differentiation of Abnormal CRs by DLAD eFigure 9. Examples of Differentiation of False-Positively Classified Normal CRs by DLAD eTable 1. Inclusion and Exclusion Criteria for the Development and External Validation Datasets eTable 2. Demographic Description of the Five External Validation Datasets eTable 3. Sensitivities of DLAD for Individual Diseases in the 5 External Validation Datasets eTable 4. Performance of Individual Non-Radiology Physician Readers eTable 5. Performance of Individual Board-Certified Radiologist Readers eTable 6. Performance of Individual Thoracic Radiologist Readers eTable 7. Performance of DLAD in the Differentiation of Abnormal CRs eTable 8. Differentiation of False-Positively Classified Normal CRs by DLAD [file jamanetwopen-2-e191095-s001.pdf]

Supplementary Online Content

Hwang EJ, Park S, Jin K-N, et al; DLAD Development and Evaluation Group. Development and validation of a deep learning–based automated detection algorithm for major thoracic diseases on chest radiographs. *JAMA Netw Open*. 2019;2(3):e191095. doi:10.1001/jamanetworkopen.2019.1095

eAppendix. Supplemental Material

eReferences

eFigure 1. Flow Diagram of Data Inclusion, Curation, and Allocation

eFigure 2. Architecture of the DLAD Algorithm

eFigure 3. User Interface of the Observer Performance Test

eFigure 4. Comparison of Performances Between Physician Only Reading and Physician Assisted by DLAD

eFigure 5. Representative Case From the Observer Performance Test (Active Pulmonary Tuberculosis)

eFigure 6. Representative Case From the Observer Performance Test (Pneumothorax)

eFigure 7. Confusion Matrices for Differentiation of Abnormal CRs

eFigure 8. Examples of the Differentiation of Abnormal CRs by DLAD

eFigure 9. Examples of Differentiation of False-Positively Classified Normal CRs by DLAD

eTable 1. Inclusion and Exclusion Criteria for the Development and External Validation Datasets

eTable 2. Demographic Description of the Five External Validation Datasets

eTable 3. Sensitivities of DLAD for Individual Diseases in the 5 External Validation Datasets

eTable 4. Performance of Individual Non-Radiology Physician Readers

eTable 5. Performance of Individual Board-Certified Radiologist Readers

eTable 6. Performance of Individual Thoracic Radiologist Readers

eTable 7. Performance of DLAD in the Differentiation of Abnormal CRs

eTable 8. Differentiation of False-Positively Classified Normal CRs by DLAD

This supplementary material has been provided by the authors to give readers additional information about their work.

eAppendix: Supplemental Material

Architecture and training process of the DLAD

Our DLAD was developed using TensorFlow (available at http://www.tensorflow.org), an open-source machine learning library from the Google Brain Team. The deep convolutional neural network used for this DLAD comprised one backbone network and five parallel classifiers. Each classifier was utilized to classify CRs of each of the four target diseases, and finally CRs with any of the target diseases. The backbone network was composed of 120 convolutional layers with four dense blocks, which utilized the CR as input, generating a feature map. The feature map was then applied to the parallel classifiers, generating probability maps for each class. The probability map highlighted the location of the abnormality in the CR, and the maximum probability value in the map was regarded as the probability value of the CR for the class.

Given an input CR with annotations for the lesion location, the loss function for each class was defined as the sum of the classification loss and localization loss. By defining the loss function as the sum of the two losses, we were able to train our network to classify the target diseases as well as determine its location. Classification loss was defined as the binary cross-entropy between the label of CR and the max-pooling of the corresponding probability map. Localization loss was defined as the average pixel-wise binary cross-entropy between the annotation on CR and the corresponding probability map. The losses of the four target disease classes were then finally summed to form the final loss function. In the case of CR inputs without annotation, only the classification loss was utilized. To predict lesion location, even without location information, we utilized a weakly-supervised localization scheme when we defined the aforementioned classification loss.[1]

In the pre-processing procedure, various image augmentation techniques such as geometric augmentations (horizontal flipping, image cropping, and image rotation) and photometric augmentations (brightness adjustment, contrast adjustment, gamma jittering, and noise injection) were applied to each of the training CRs before being fed into the network. These augmentations helped the network perform robustly against various lesion sizes, geometries, imaging conditions, and equipment.

All of the trainable parameters were initialized randomly via Gaussian distribution. All models were trained using the stochastic gradient descent (SGD) with a mini-batch size of 64. We also applied a learning rate of 0.01 and a momentum term of 0.9 to stabilize the training. We then decreased the learning rate from 0.01 to 0.001 after 30 epochs. The models were trained up to 40 epochs. For further improvement of its generalizability, three

© 2019 Hwang EJ et al. *JAMA Network Open.*

networks using the same data but different hyper-parameters were independently trained and the resultant predictions were averaged to make the final prediction.[2]

Anyone who would like to test our DLAD can upload their own DICOM files as input and check the output results of the DLAD on our website (http://insights.lunit.io/) for free.

eReferences

1. Naftaly U, Intrator N, Horn D. Optimal ensemble averaging of neural networks. *Network-Comp Neural.* 1997;8(3):283-296.
2. Pinheiro PO, Collobert R. From image-level to pixel-level labeling with convolutional networks. Paper presented at: Proceedings of the IEEE Conference on Computer Vision and Pattern Recognition2015.

© 2019 Hwang EJ et al. *JAMA Network Open.*

eFigure 1. Flow diagram of data inclusion, curation, and allocation

Initially, 98,621 chest radiographs [CRs] (57,481 normal CRs and 41,140 abnormal CRs) were included and underwent the data curation process including labeling and annotation. A total of 8,787 CRs (3,260 normal CRs and 5,527 abnormal CRs) were excluded during the data curation process and the remaining 89,834 CRs (54,221 normal CRs and 35,613 abnormal CRs) were assigned into one of three datasets: training dataset, tuning dataset, or an in-house validation dataset.

© 2019 Hwang EJ et al. *JAMA Network Open.*

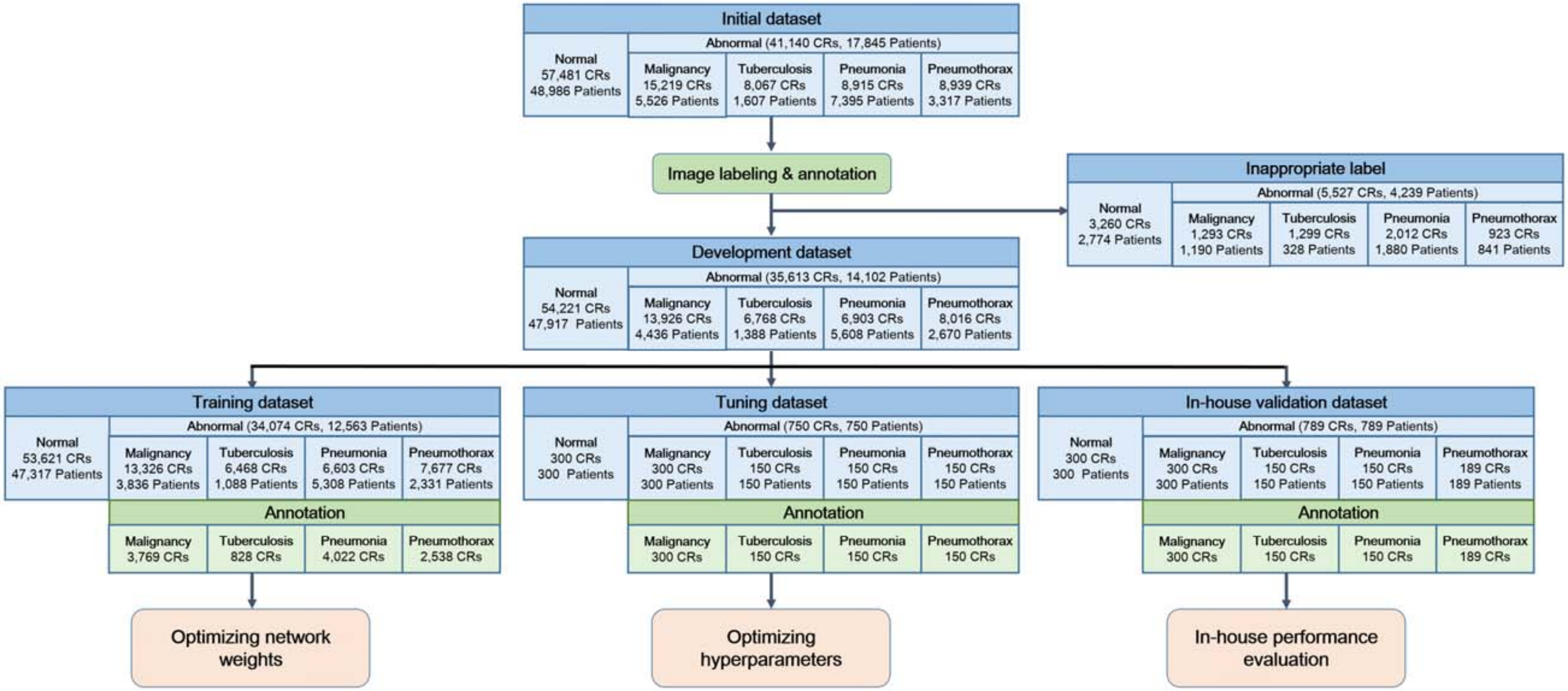

© 2019 Hwang EJ et al. *JAMA Network Open.*

eFigure 2. Architecture of the DLAD algorithm

The deep convolutional neural network used for DLAD comprised one backbone network and five parallel classifiers. Four classifiers were designed for each disease, and the final classifier was for the classification of CRs with any of the target diseases. The backbone network was composed of 120 convolutional layers with four dense blocks, which utilized the CR as input, generating a feature map. The feature map was then applied to the parallel classifiers, generating probability maps for each class.

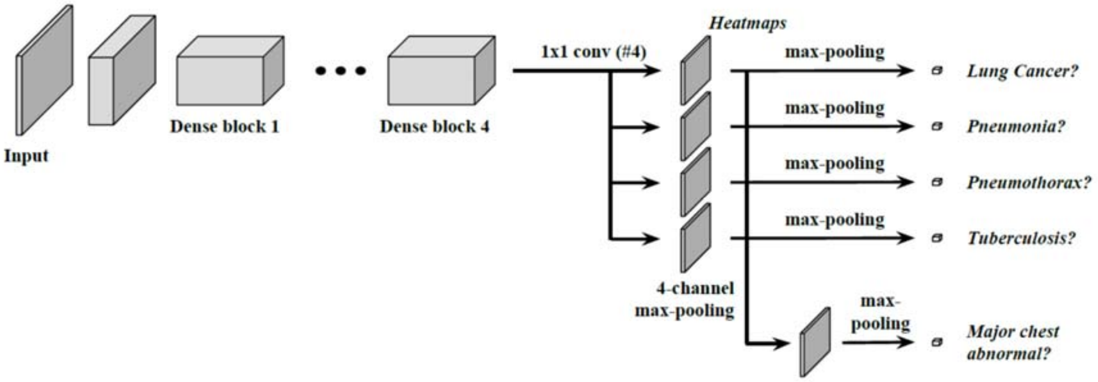

© 2019 Hwang EJ et al. *JAMA Network Open.*

eFigure 3. User interface of the observer performance test

A web-based user interface was utilized for the observer performance test. In session 1, observers were provided only a single CR image, without any additional information. The user interface provided basic image adjustment functions, including zooming, panning, inverting, and adjusting of the window level and width (A). Each observer was first asked to determine whether there was any clinically significant abnormal findings requiring treatment or further evaluation. If the observer classified the CR as an abnormal CR, the observer was then asked to annotate the location of the abnormality using free-hand annotation. For each annotation, observers provided a confidence score with a continuous value between 0 and 1, and a brief description of the abnormality (B). After saving the annotation and confidence score, observers could then proceed to session 2 (C). In session 2, observers were additionally provided the output of the deep-learning assisted detection algorithm (DLAD) for the presence of any of the target diseases. Observers were able to find the image-wise probability value and overlay the probability map on the CR. After reviewing the DLAD's output and the observer's own answers from session 1, the observers were asked to modify their initial answer, including the presence and location of the abnormality, as well as the confidence score for each annotation (D).

© 2019 Hwang EJ et al. *JAMA Network Open.*

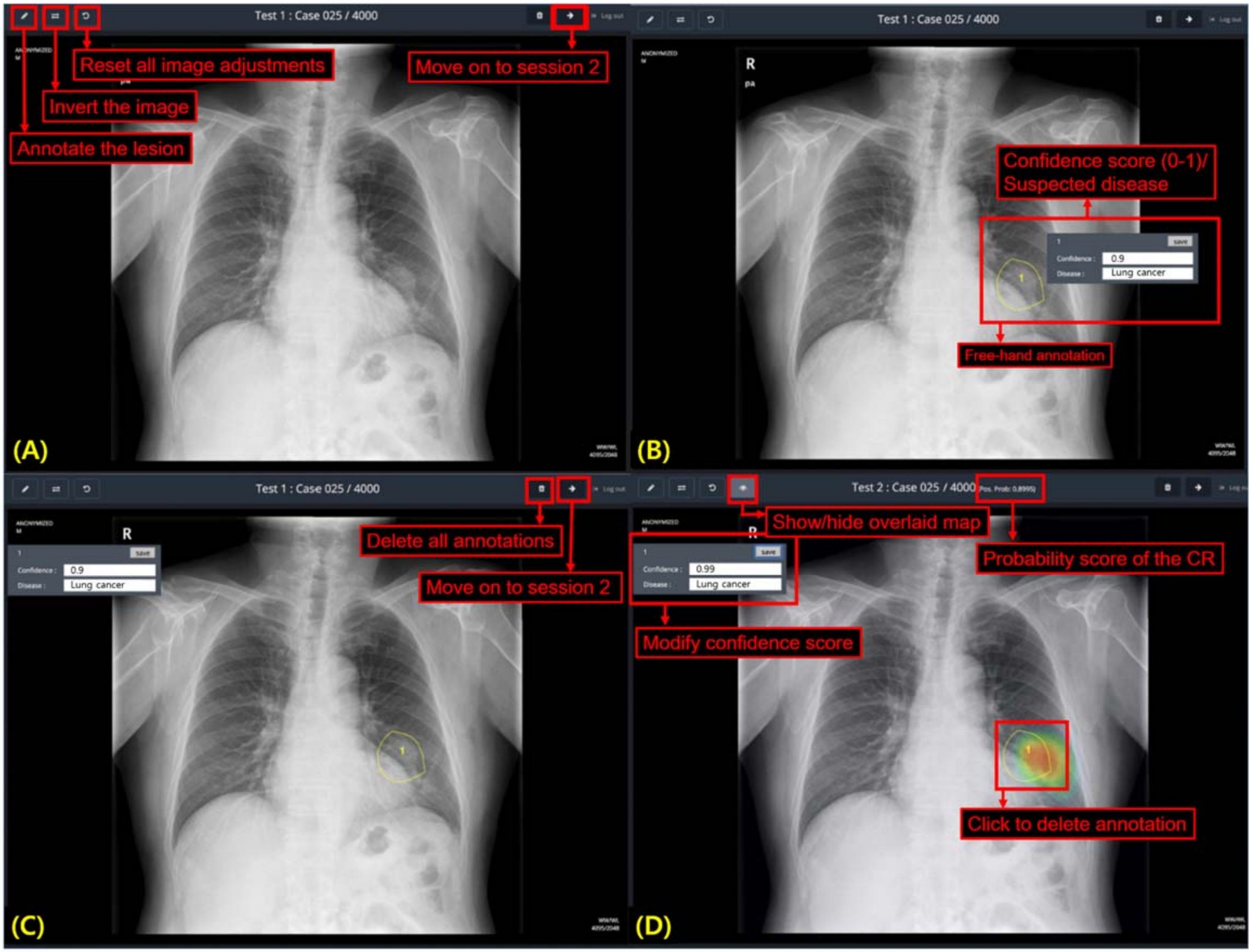

© 2019 Hwang EJ et al. *JAMA Network Open.*

eFigure 4. Comparison of performances between physician only reading and physician assisted by DLAD

There were significant improvements in all three observer groups when assisted by DLAD, not only in image-wise classification performance (non-radiology physicians [area under the receiver operating characteristic curves 0.814 to 0.904]; board-certified radiologists [0.896 to 0.939]; and thoracic radiologists [0.932 to 0.958]), but also in lesion-wise localization performance (non-radiology physicians [area under the alternative free-response receiver operating characteristic curves 0.781 to 0.873]; board-certified radiologists [0.870 to 0.919]; and thoracic radiologists [0.907 to 0.938]).

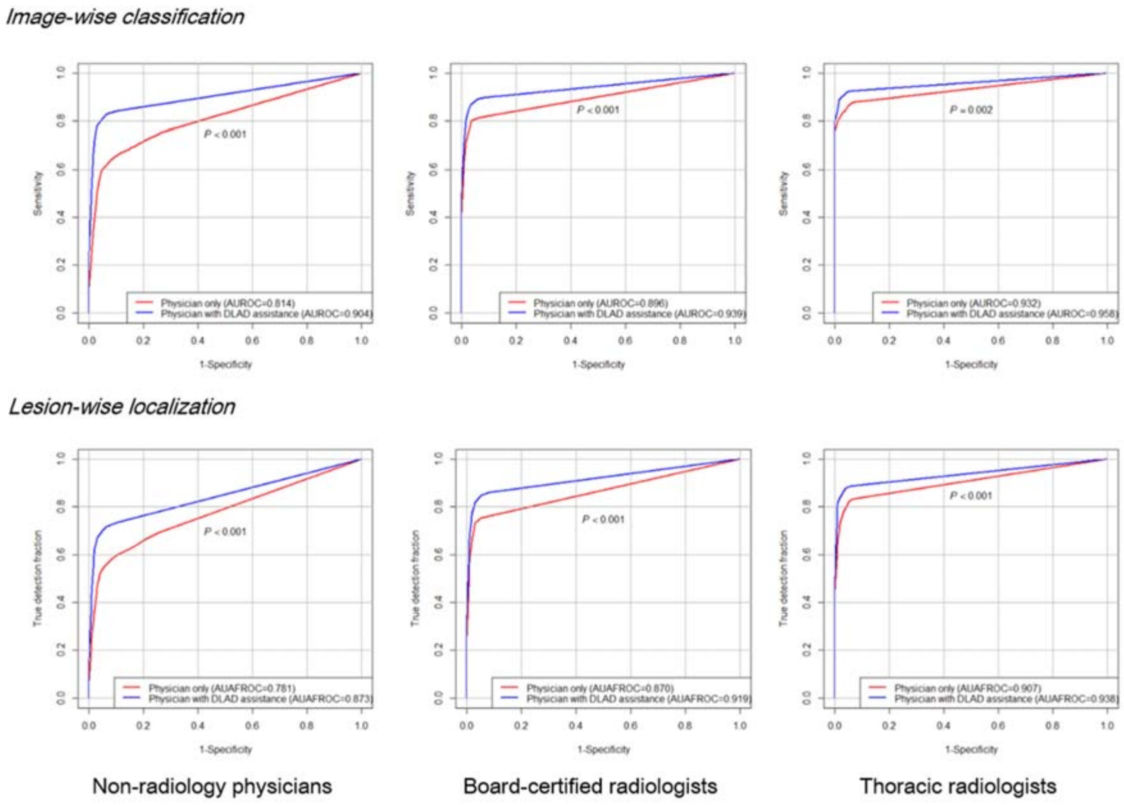

© 2019 Hwang EJ et al. *JAMA Network Open.*

eFigure 5. Representative case from the observer performance test (active pulmonary tuberculosis)

CR (left) of a patient with active pulmonary tuberculosis shows a patchy increased opacity at the retrocardiac area, which was initially detected by seven out of 15 observers (four thoracic radiologists and three board-certified radiologists.) The corresponding CT image (middle) demonstrated a cavitary consolidation at the left lower lobe, which was confirmed as active pulmonary tuberculosis. DLAD correctly localized this lesion with a probability value of 0.299 (right). After checking the DLAD's output, five physicians (one thoracic radiologist, two board-certified radiologists, and two non-radiology physicians) additionally detected the lesion.

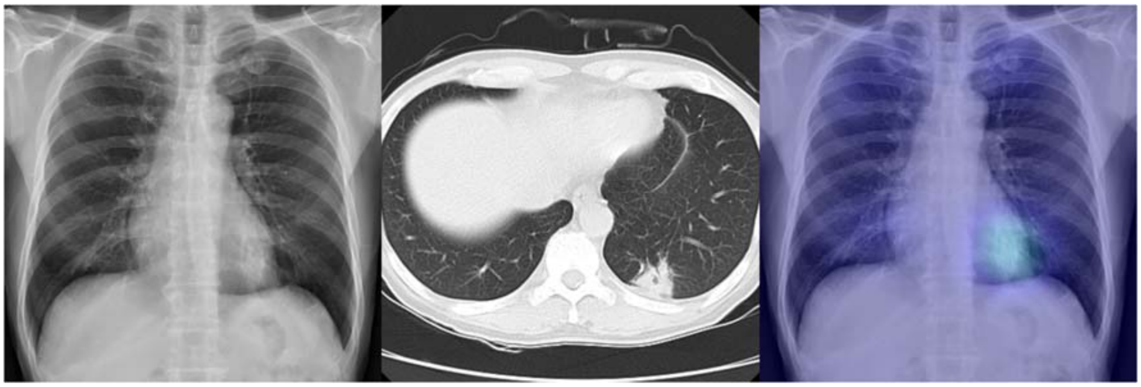

© 2019 Hwang EJ et al. *JAMA Network Open.*

eFigure 6. Representative case from the observer performance test (pneumothorax)

CR (left) demonstrates a small amount of pneumothorax in the left hemithorax, which was initially missed by four out of 15 physicians (one thoracic radiologist, one board-certified radiologists, and two non-radiology physicians). DLAD correctly localized the pneumothorax with a probability value of 0.463 (right). After checking the DLAD result, only one physician (non-radiology physician) misclassified the CR.

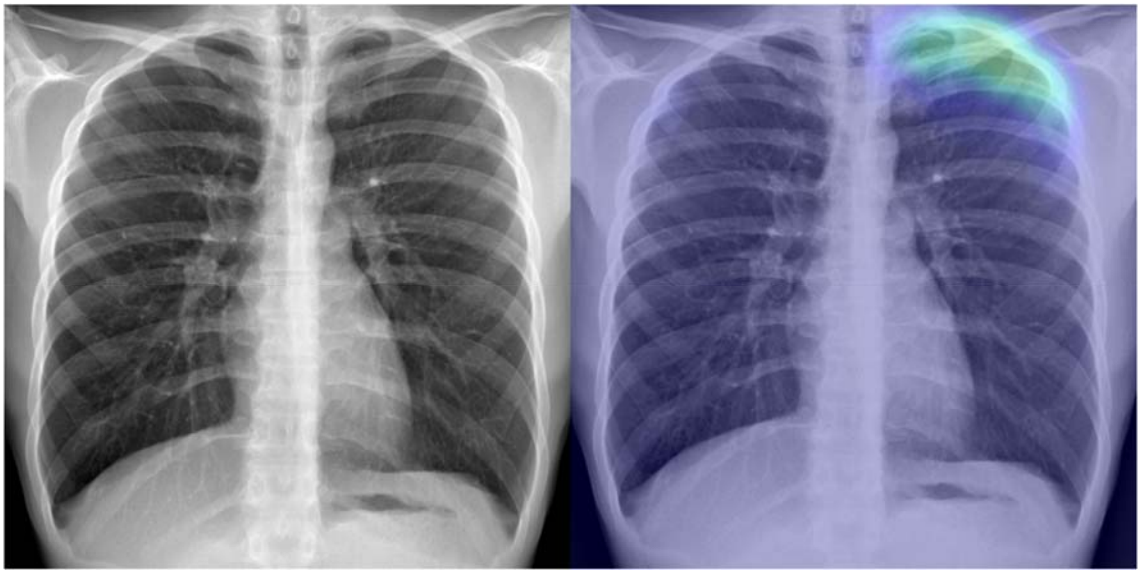

© 2019 Hwang EJ et al. *JAMA Network Open.*

eFigure 7. Confusion matrices for differentiation of abnormal CRs

Confusion matrices show the number of CRs for a combination between the reference standard diagnosis and the differential diagnosis of DLAD in the pooled external validation datasets and each external validation dataset. Numbers of accurate differential diagnoses are shown in diagonal. The pooled overall accuracy was 0.686, ranging from 0.649 to 0.714, among institutions.

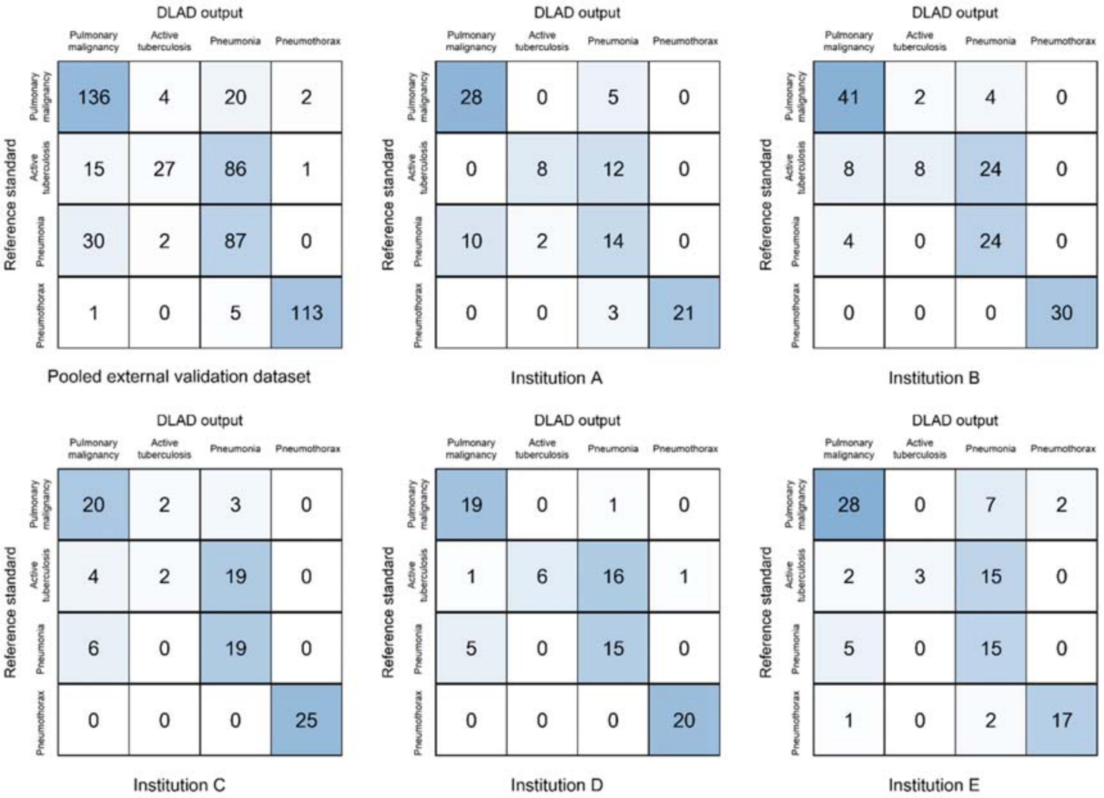

© 2019 Hwang EJ et al. *JAMA Network Open.*

eFigure 8. Examples of the differentiation of abnormal CRs by DLAD

Images in the first column show the original CR images and images in the next four columns show the probability maps from DLAD in classifying CRs with pulmonary malignancy, active tuberculosis, pneumonia, and pneumothorax. The upper four cases show examples of accurate differentiation, while the lower three cases show examples of missed differentiation. The reference standard diagnosis of the fifth case is pneumonia, however, DLAD classified the CR as a pulmonary malignancy. The original CR image shows nodular consolidation at the left lower lung field, mimicking lung cancer. The reference standard diagnosis of the sixth case is active tuberculosis, however, DLAD classified it as pneumonia. The original CR image also shows consolidation in the right upper lobe, mimicking pneumonia. The reference standard diagnosis of the last case is active tuberculosis, however, DLAD classified it as pulmonary malignancy. In the original CR image, there is a nodular opacity at the retrocardiac left lung, mimicking lung cancer.

© 2019 Hwang EJ et al. *JAMA Network Open.*

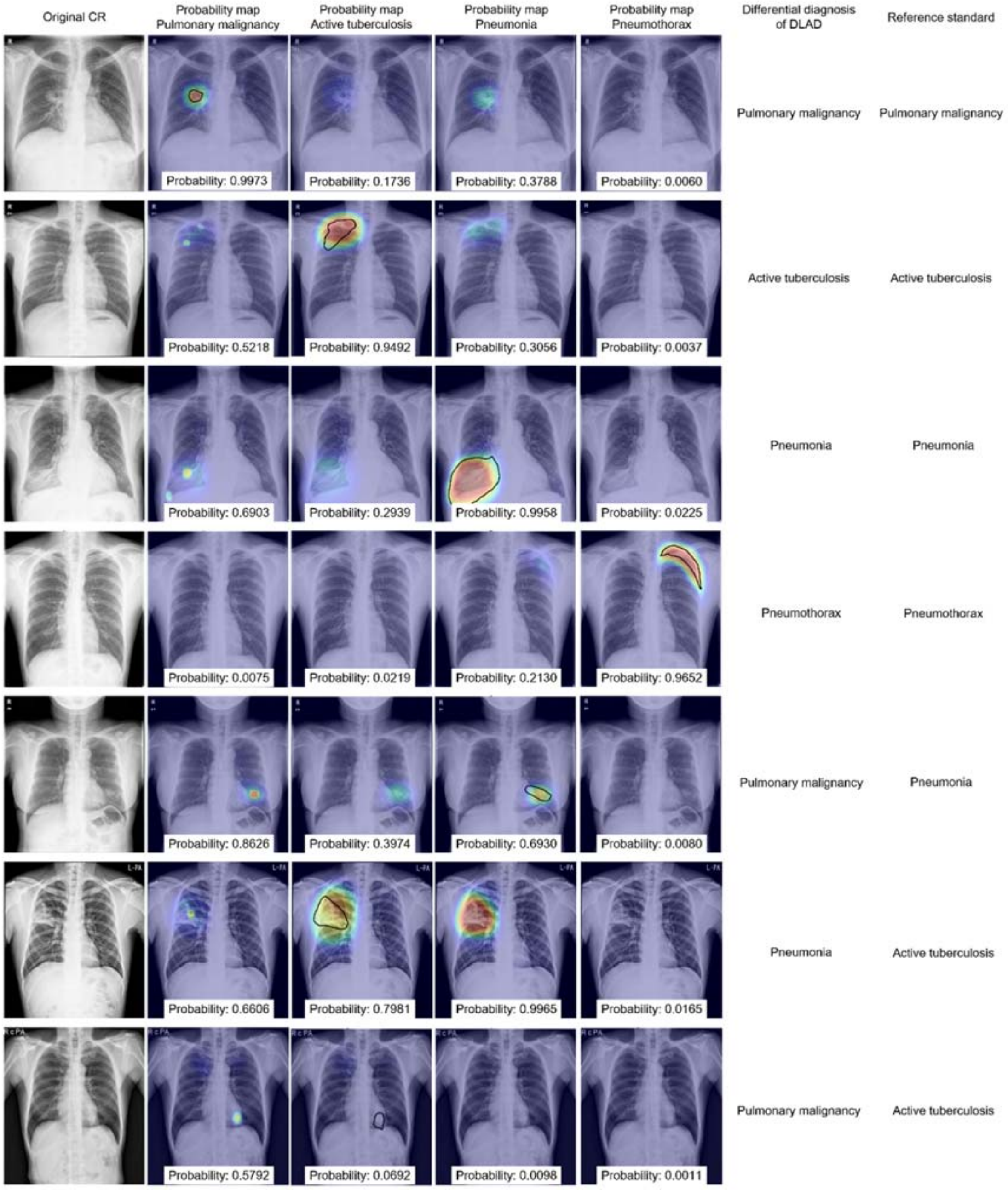

© 2019 Hwang EJ et al. *JAMA Network Open.*

eFigure 9. Examples of differentiation of false-positively classified normal CRs by DLAD

The majority of normal CRs falsely designated as abnormal by DLAD were falsely designated as having pulmonary malignancies (87.2% [82/94] at high sensitivity threshold, and 89.3% [25/28] at high specificity threshold). The figure shows example of three false positive cases that DLAD categorized CRs as having pulmonary malignancies. Images in the first column show the original CR images, while images in the next five columns show the probability maps provided by DLAD in classifying CRs with any of major thoracic diseases, pulmonary malignancy, active tuberculosis, pneumonia, and pneumothorax, respectively. In the case at the first row, the DLAD misdiagnosed right nipple as a small pulmonary nodule. In the case at the second row, the DLAD classified the CR as abnormal CR and designated it as CR with pulmonary malignancy, focal increased opacity caused by overlapping ribs was misdiagnosed as pulmonary nodule. In the case at the third row, the DLAD misdiagnosed the right hilar pulmonary artery as a pulmonary nodule. It is interesting that focal lesions that are frequently misdiagnosed by inexperienced physicians were similar with those misdiagnosed by the DLAD.

© 2019 Hwang EJ et al. *JAMA Network Open.*

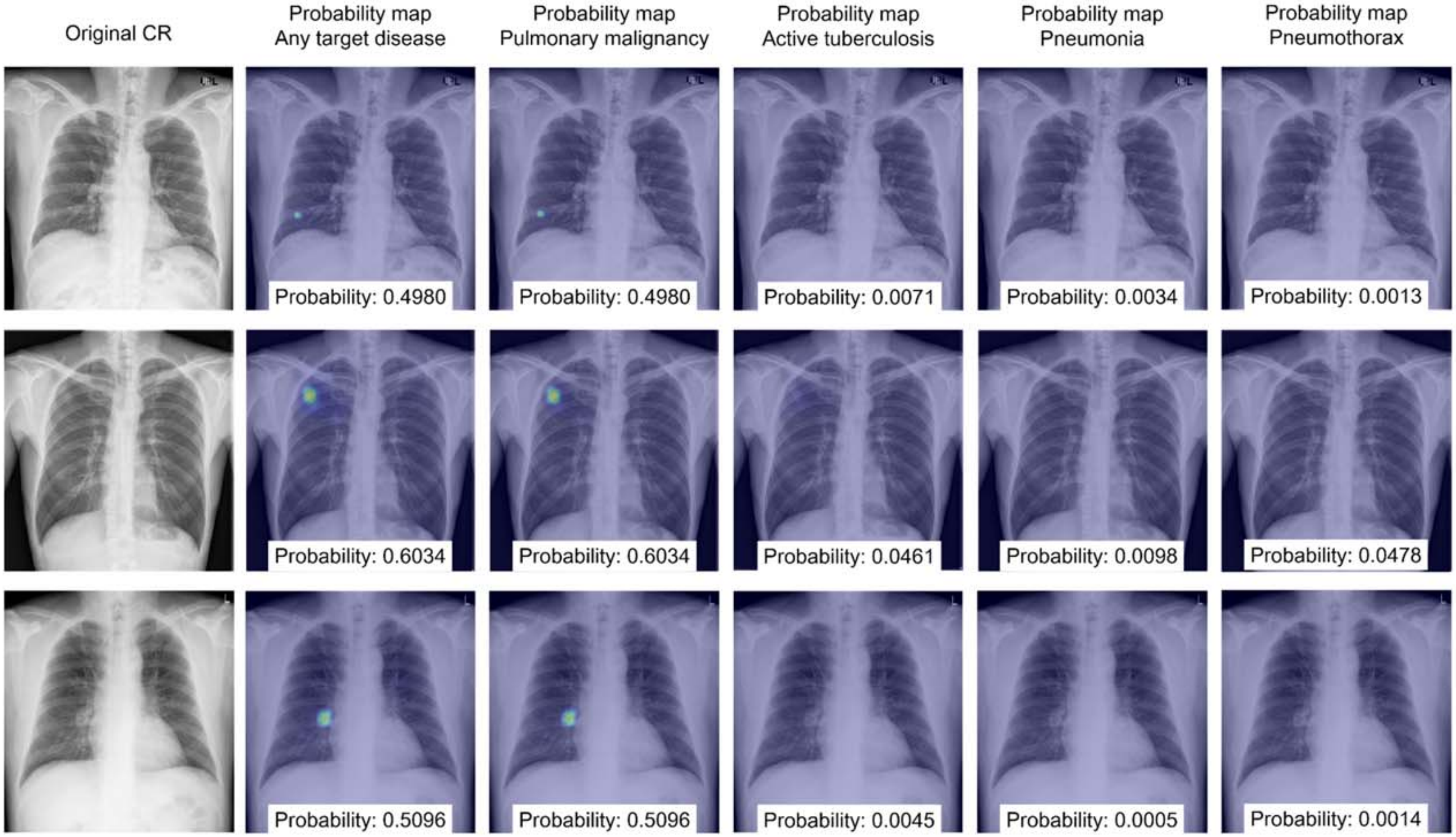

© 2019 Hwang EJ et al. *JAMA Network Open.*

eTable 1. Inclusion and exclusion criteria for the development and external validation datasets

Development dataset
Normal CRs<br>*Inclusion criteria*<br>CRs taken between 2010 and 2015.<br>CRs reported as normal by radiologists.
Abnormal CRs
Pulmonary malignancies<br>*Inclusion criteria*<br>CRs taken between 2010 and 2015.<br>CRs from patients with pathologically diagnosed pulmonary malignancies.<br>CRs taken within 1 month from the date of diagnosis.
Active pulmonary tuberculosis<br>*Inclusion criteria*<br>CRs taken between 2010 and 2015.<br>CRs from patients with active pulmonary tuberculosis diagnosed via culture or PCR.<br>CRs taken within 2 weeks from the date of initial treatment.
Pneumonia<br>*Inclusion criteria*<br>CRs taken between 2010 and 2015.<br>CRs from patients with microbiologically or clinically diagnosed pneumonia.<br>CRs taken within 1 week from the date of diagnosis.
Pneumothorax<br>*Inclusion criteria*<br>CRs taken between 2010 and 2015.<br>CRs with mention of pneumothorax in the report.
External validation datasets
Normal CRs<br>*Inclusion criteria*<br>CRs from patients with no referable abnormality on chest CT.<br>CRs taken within 2 weeks from the chest CT.<br>(For Institution A) CRs taken between September and October 2017.
Abnormal CRs
Pulmonary malignancies<br>*Inclusion criteria*<br>CRs from patients with pathologically or clinically diagnosed pulmonary malignancies.<br>CRs with corresponding chest CT taken within 1 month.<br>(For Institution A) CRs taken between December 2016 and October 2017.<br>*Exclusion criteria*<br>CRs without visible lesions.<br>CRs with >3 lesions.<br>CRs with other clinically relevant abnormalities.
Active pulmonary tuberculosis<br>*Inclusion criteria*<br>CRs from patients with active pulmonary tuberculosis diagnosed via culture or PCR.<br>CRs taken within 2 weeks from the date of initial treatment.<br>CRs with corresponding chest CT taken within 1 month.<br>(For Institution A) CRs taken between March 2017 and September 2017.<br>*Exclusion criteria*<br>CRs without visible abnormality.<br>CRs with other clinically relevant abnormalities.
Pneumonia<br>*Inclusion criteria*<br>CRs from patients with microbiologically or clinically diagnosed pneumonia.<br>CRs taken within 1 week from the date of diagnosis.<br>CRs with corresponding chest CT taken within 1 week.

© 2019 Hwang EJ et al. *JAMA Network Open.*

(For Institution A) CRs taken between March 2016 and September 2017.<br>*Exclusion criteria*
External validation datasets (continues)
CRs without visible abnormality.<br>CRs with other clinically relevant abnormalities.
Pneumothorax<br>*Inclusion criteria*<br>CRs with an unequivocal finding of pneumothorax.<br>(For Institution A) CRs taken between April 2016 and August 2017.<br>*Exclusion criteria*<br>CRs with drainage catheter or subcutaneous emphysema.<br>CRs with other clinically relevant abnormalities.<br>CRs taken immediately after thoracic surgery.

Abbreviation: CR, chest radiograph; CT, computed tomography; PCR, polymerase chain reaction

eTable 2. Demographic description of the five external validation datasets

Demographic information	Institution A	Institution B	Institution C	Institution D	Institution E
Whole CRs					
Number of patients (Male:Female)	200 (113:87)	245 (158:87)	190 (110:80)	184 (97:87)	196
Age (years)	54.2 ± 15.8	55.5 ± 17.9	51.0 ± 19.4	49.0 ± 18.7	56.8 ± 16.4
Normal CRs					
Number of patients (Male:Female)	97 (62:35)	100 (68:32)	90 (41:49)	100 (45:55)	99 (82:17)
Age (years)	53.0 ± 10.8	47.4 ± 10.7	48.6 ± 14.6	46.1 ± 18.0	61.9 ± 11.3
Abnormal CRs					
Number of patients (Male:Female)	103 (51:52)	145 (90:55)	100 (69:31)	84 (52:32)	97 (68:29)
Age (years)	55.2 ± 19.4	61.1 ± 19.6	52.4 ± 21.8	50.0 ± 18.9	51.5 ± 19.4
Pulmonary malignancies					
Number of patients (Male:Female)	33 (17:16)	47 (35:12)	25 (16:9)	20 (11:9)	37 (27:10)
Age (years)	65.6 ± 10.9	70.1 ± 10.9	66.8 ± 8.4	64.6 ± 14.5	65.8 ± 11.8
Number of lesions	36	52	26	36	40
Lesion size (cm)	2.96 ± 1.35	3.8 ± 1.7	2.30 ± 0.81	2.24 ± 0.73	4.3 ± 3.1
Proportion of obscured lesions*	33.3%	13.5%	19.2%	5.6%	10.0%
Active pulmonary tuberculosis					
Number of patients (Male:Female)	20 (12:8)	40 (11:29)	25 (14:11)	24 (17:7)	20 (14:6)
Age (years)	48.1 ± 10.9	60.4 ± 21.2	53.6 ± 20.8	51.8 ± 16.9	38.9 ± 14.9
Pneumonia					
Number of patients (Male:Female)	26 (7:19)	28 (18:10)	25 (19:6)	20 (6:14)	20 (14:6)
Age (years)	63.6 ± 15.1	68.6 ± 17.0	62.3 ± 15.3	48.2 ± 17.7	47.6 ± 19.0
Pneumothorax					
Number of patients (Male:Female)	24 (15:9)	30 (26:4)	25 (20:5)	20 (18:2)	20 (13:7)
Age (years)	37.8 ± 19.8	42.6 ± 19.4	27.0 ± 14.3	28.1 ± 14.7	41.9 ± 19.4
Proportion of large pneumothorax†	20.8%	93.3%	60%	65%	40%

Abbreviations: CR, chest radiograph

Data are mean ± standard deviation.

*Lesions partly or totally obscured by the heart, diaphragm, or hilar vessels.

†Interpleural distance at the level of the hilum >2 cm (British Thoracic Society guideline).

© 2019 Hwang EJ et al. *JAMA Network Open.*

eTable3. Sensitivities of DLAD for individual diseases in the 5 external validation datasets

Disease	Institution A	Institution B	Institution C	Institution D	Institution E
High sensitivity threshold					
Malignant nodule	0.909 (0.757 – 0.981)	1.000 (0.925 – 1.000)	1.000 (0.863 – 1.000)	1.000 (0.832 – 1.000)	1.000 (0.905 – 1.000)
Active tuberculosis	1.000 (0.832 – 1.000)	0.925 (0.796 – 0.984)	1.000 (0.863 – 1.000)	1.000 (0.858 – 1.000)	1.000 (0.832 – 1.000)
Pneumonia	0.923 (0.749 – 0.991)	1.000 (0.877 – 1.000)	1.000 (0.863 – 1.000)	1.000 (0.832 – 1.000)	0.950 (0.751 – 0.999)
Pneumothorax	0.833 (0.626 – 0.953)	1.000 (0.884 – 1.000)	1.000 (0.863 – 1.000)	1.000 (0.832 – 1.000)	0.950 (0.751 – 0.999)
High specificity threshold					
Malignant nodule	0.818 (0.645 – 0.930)	0.936 (0.825 – 0.987)	0.920 (0.740 – 0.990)	1.000 (0.832 – 1.000)	0.946 (0.818 – 0.993)
Active tuberculosis	0.950 (0.751 – 0.999)	0.925 (0.796 – 0.984)	0.960 (0.796 – 0.999)	1.000 (0.858 – 1.000)	1.000 (0.832 – 1.000)
Pneumonia	0.808 (0.606 – 0.934)	0.964 (0.816 – 0.999)	1.000 (0.863 – 1.000)	1.000 (0.832 – 1.000)	0.850 (0.621 – 0.968)
Pneumothorax	0.833 (0.626 – 0.953)	1.000 (0.884 – 1.000)	1.000 (0.863 – 1.000)	1.000 (0.832 – 1.000)	0.850 (0.621 – 0.968)

Abbreviations: AUROC, area under the receiver operating characteristic curve; AUAFROC, area under the alternative free-response receiver operating characteristic curve

© 2019 Hwang EJ et al. *JAMA Network Open.*

eTable 4. Performance of individual non-radiology physician readers

Observer	AUROC	*P*-value	AUAFROC	*P*-value	Sensitivity	*P*-value	Specificity	*P*-value
Session 1 (Physician without DLAD assistance)								
Non-radiology physician 1	0.779 (0.729 – 0.830)	<.001*	0.763 (0.714 – 0.812)	<.001*	0.582 (0.481 – 0.679)		0.969 (0.912 – 0.994)	
Non-radiology physician 2	0.868 (0.823 – 0.914)	<.001*	0.844 (0.800 – 0.888)	<.001*	0.777 (0.684 – 0.853)		0.948 (0.884 – 0.983)	
Non-radiology physician 3	0.816 (0.766 – 0.866)	<.001*	0.780 (0.731 – 0.829)	<.001*	0.670 (0.570 – 0.759)		0.959 (0.898 – 0.989)	
Non-radiology physician 4	0.823 (0.767 – 0.879)	<.001*	0.778 (0.723 – 0.833)	<.001*	0.816 (0.727 – 0.885)		0.742 (0.643 – 0.826)	
Non-radiology physician 5	0.782 (0.727 – 0.837)	<.001*	0.742 (0.689 – 0.796)	<.001*	0.650 (0.550 – 0.742)		0.887 (0.806 – 0.942)	
Session 2 (Physician with DLAD assistance)								
Non-radiology physician 1	0.850 (0.805 – 0.894)	<.001†	0.836 (0.793 – 0.879)	<.001†	0.699 (0.601 – 0.785)	.002†	1.000 (0.963 – 1.000)	.248†
Non-radiology physician 2	0.964 (0.938 – 0.990)	<.001†	0.952 (0.924 – 0.979)	<.001†	0.942 (0.878 – 0.978)	<.001†	0.979 (0.927 – 0.997)	.248†
Non-radiology physician 3	0.896 (0.856 – 0.936)	<.001†	0.849 (0.808 – 0.889)	<.001†	0.806 (0.716 – 0.877)	.001†	0.969 (0.912 – 0.994)	1.000†
Non-radiology physician 4	0.919 (0.879 – 0.960)	<.001†	0.881 (0.837 – 0.925)	<.001†	0.903 (0.829 – 0.952)	.008†	0.742 (0.643 – 0.826)	1.000†
Non-radiology physician 5	0.893 (0.850 – 0.935)	<.001†	0.847 (0.804 – 0.890)	<.001†	0.825 (0.738 – 0.893)	<.001†	0.928 (0.857 – 0.970)	.134†

Numbers in parentheses indicate the 95% confidence interval.

Abbreviations: AUROC, area under the receiver operating characteristic curve; AUAFROC, area under the alternative free-response receiver operating characteristic curve; DLAD, deep-learning based automatic detection algorithm

*Comparison of performance with DLAD.

†Comparison of performance with session 1.

© 2019 Hwang EJ et al. *JAMA Network Open.*

eTable 5. Performance of individual board-certified radiologist readers

Observer	AUROC	*P*-value	AUAFROC	*P*-value	Sensitivity	*P*-value	Specificity	*P*-value
Session 1 (Physician without DLAD assistance)								
Board-certified radiologist 1	0.915 (0.877 – 0.954)	<.001*	0.888 (0.849 – 0.927)	<.001*	0.845 (0.760 – 0.909)		0.938 (0.870 – 0.977)	
Board-certified radiologist 2	0.911 (0.872 – 0.949)	<.001*	0.877 (0.838 – 0.916)	<.001*	0.835 (0.749 – 0.901)		0.948 (0.884 – 0.983)	
Board-certified radiologist 3	0.856 (0.810 – 0.901)	<.001*	0.831 (0.786 – 0.876)	<.001*	0.728 (0.632 – 0.811)		0.969 (0.912 – 0.994)	
Board-certified radiologist 4	0.913 (0.873 – 0.953)	<.001*	0.889 (0.847 – 0.932)	<.001*	0.864 (0.782 – 0.924)		0.918 (0.844 – 0.964)	
Board-certified radiologist 5	0.887 (0.845 – 0.929)	<.001*	0.865 (0.824 – 0.907)	<.001*	0.786 (0.695 – 0.861)		0.969 (0.912 – 0.994)	
Session 2 (Physician with DLAD assistance)								
Board-certified radiologist 1	0.951 (0.921 – 0.981)	.007†	0.944 (0.915 – 0.973)	<.001†	0.913 (0.841 – 0.959)	.023†	0.918 (0.844 – 0.964)	.480†
Board-certified radiologist 2	0.938 (0.905 – 0.972)	.015†	0.909 (0.873 – 0.945)	.004†	0.884 (0.805 – 0.938)	.074†	0.948 (0.884 – 0.983)	1.000†
Board-certified radiologist 3	0.920 (0.884 – 0.957)	<.001†	0.890 (0.853 – 0.928)	<.001†	0.854 (0.771 – 0.916)	.001†	0.979 (0.927 – 0.997)	1.000†
Board-certified radiologist 4	0.953 (0.922 – 0.983)	.003†	0.935 (0.902 – 0.968)	.001†	0.942 (0.878 – 0.978)	.013†	0.918 (0.844 – 0.964)	1.000†
Board-certified radiologist 5	0.935 0.902 – 0.968)	.001†	0.916 (0.883 – 0.949)	<.001†	0.874 (0.794 – 0.931)	.008†	0.979 (0.927 – 0.997)	1.000†

Numbers in parentheses indicate the 95% confidence interval.

Abbreviations: AUROC, area under the receiver operating characteristic curve; AUAFROC, area under the alternative free-response receiver operating characteristic curve; DLAD, deep-learning based automatic detection algorithm

*Comparison of performance with DLAD.

†Comparison of performance with session 1.

© 2019 Hwang EJ et al. *JAMA Network Open.*

eTable 6. Performance of individual thoracic radiologist readers

Observer	AUROC	*P*-value	AUAFROC	*P*-value	Sensitivity	*P*-value	Specificity	*P*-value
Session 1 (Physician without DLAD assistance)								
Thoracic radiologist 1	0.920 (0.883 – 0.957)	<.001[*]	0.892 (0.853 – 0.932)	<.001[*]	0.854 (0.771 – 0.916)		0.938 (0.870 – 0.977)	
Thoracic radiologist 2	0.933 (0.899 – 0.967)	.010[*]	0.919 (0.884 – 0.955)	<.001[*]	0.884 (0.805 – 0.938)		0.928 (0.857 – 0.970)	
Thoracic radiologist 3	0.906 (0.867 – 0.945)	<.001[*]	0.881 (0.840 – 0.921)	<.001[*]	0.825 (0.737 – 0.893)		0.948 (0.884 – 0.983)	
Thoracic radiologist 4	0.941 (0.909 – 0.973)	.006[*]	0.905 (0.870 – 0.939)	<.001[*]	0.884 (0.805 – 0.938)		0.990 (0.944 – 1.000)	
Thoracic radiologist 5	0.959 (0.932 – 0.986)	.140[*]	0.938 (0.909 – 0.968)	.005[*]	0.932 (0.865 – 0.972)		0.928 (0.857 – 0.970)	
Session 2 (Physician with DLAD assistance)								
Thoracic radiologist 1	0.956 (0.928 – 0.984)	.007[†]	0.927 (0.894 – 0.960)	.004[†]	0.922 (0.853 – 0.966)	.023[†]	0.938 (0.870 – 0.977)	1.000[†]
Thoracic radiologist 2	0.966 (0.942 – 0.991)	.007[†]	0.953 (0.927 – 0.979)	.008[†]	0.942 (0.878 – 0.978)	.041[†]	0.938 (0.870 – 0.977)	1.000[†]
Thoracic radiologist 3	0.934 (0.900 – 0.968)	.015[†]	0.922 (0.886 – 0.959)	.001[†]	0.874 (0.794 – 0.931)	.074[†]	0.959 (0.898 – 0.989)	1.000[†]
Thoracic radiologist 4	0.970 (0.947 – 0.993)	.014[†]	0.933 (0.904 – 0.962)	.022[†]	0.942 (0.878 – 0.978)	.041[†]	0.979 (0.927 – 0.997)	1.000[†]
Thoracic radiologist 5	0.966 (0.941 – 0.991)	.204[†]	0.953 (0.926 – 0.979)	.054[†]	0.942 (0.878 – 0.978)	1.000[†]	0.928 (0.857 – 0.970)	1.000[†]

Numbers in parentheses indicate the 95% confidence interval.

Abbreviations: AUROC, area under the receiver operating characteristic curve; AUAFROC, area under the alternative free-response receiver operating characteristic curve; DLAD, deep-learning based automatic detection algorithm

[*]Comparison of performance with DLAD.

[†]Comparison of performance with session 1.

© 2019 Hwang EJ et al. *JAMA Network Open.*

eTable 7. Performance of DLAD in the differentiation of abnormal CRs

Disease	Institution A	Institution B	Institution C	Institution D	Institution E
Overall accuracy	0.689 (0.591 – 0.777)	0.710 (0.629 – 0.783)	0.660 (0.558 – 0.752)	0.714 (0.605 – 0.808)	0.649 (0.546 – 0.744)
Producer's accuracies for each target disease					
Pulmonary malignancies	0.848 (0.681 – 0.949)	0.872 (0.743 – 0.952)	0.800 (0.593 – 0.932)	0.950 (0.751 – 0.999)	0.757 (0.588 – 0.882)
Active tuberculosis	0.400 (0.191 – 0.639)	0.200 (0.091 – 0.356)	0.080 (0.010 – 0.260)	0.250 (0.098 – 0.467)	0.150 (0.032 – 0.379)
Pneumonia	0.538 (0.334 – 0.734)	0.857 (0.673 – 0.960)	0.760 (0.549 – 0.906)	0.750 (0.509 – 0.913)	0.750 (0.509 – 0.913)
Pneumothorax	0.875 (0.676 – 0.973)	1.000 (0.884 – 1.000)	1.000 (0.863 – 1.000)	1.000 (0.832 – 1.000)	0.850 (0.621 – 0.968)

Numbers in parentheses indicate the 95% confidence interval

Abbreviations: DLAD, deep-learning based automatic detection algorithm; CR, Chest radiograph

© 2019 Hwang EJ et al. *JAMA Network Open.*

eTable 8. Differentiation of false-positively classified normal CRs by DLAD

DLAD's differentiation	Institution A	Institution B	Institution C	Institution D	Institution E	Whole external validation dataset
High sensitivity threshold						
Malignant nodule	0	12 (100%)	27 (81.8%)	5 (83.3%)	38 (88.4%)	82 (87.2%)
Active tuberculosis	0	0	4 (12.1%)	0	0	4 (4.3%)
Pneumonia	0	0	0	1 (16.7%)	5 (11.6%)	6 (6.4%)
Pneumothorax	0	0	2 (6.1%)	0	0	2 (2.1%)
Sum	0	12 (100%)	33 (100%)	6 (100%)	43 (100%)	94 (100%)
High specificity threshold						
Malignant nodule	0	2 (100%)	10 (90.9%)	0	13 (86.7%)	25 (89.3%)
Active tuberculosis	0	0	1 (9.1%)	0	0	1 (3.6%)
Pneumonia	0	0	0	0	2 (13.3%)	2 (7.1%)
Pneumothorax	0	0	0	0	0	0
Sum	0	2 (100%)	11 (100%)	0	15 (100%)	28 (100%)

Numbers indicate numbers of CRs differentiated as each category by DLAD.

Numbers in parentheses indicate proportions of CRs differentiated as each category, among all false-positively classified CRs.

Abbreviations: DLAD, deep-learning based automatic detection algorithm

© 2019 Hwang EJ et al. *JAMA Network Open.*
